# 1-Acetyl-3-(4-chloro­phen­yl)-5-(4-fluoro­phen­yl)-2-pyrazoline

**DOI:** 10.1107/S1600536808018333

**Published:** 2008-07-05

**Authors:** Jian Li, Huai-Fen Xiao, Jie Yang

**Affiliations:** a Department of Educational Science and Technology, Weifang University, Weifang 261061, People’s Republic of China; b Experimental Junior Middle School of Changle County, Weifang 261061, People’s Republic of China

## Abstract

In the title mol­ecule, C_17_H_14_ClFN_2_O, the mean plane of the pyrazoline ring makes dihedral angles of 18.19 (1) and 83.51 (4)° with the 4-chloro­benzene and 4-fluoro­benzene rings, respectively. The two benzene rings make a dihedral angle of 76.11 (2)°. Weak inter­molecular C—H⋯O hydrogen bonds help stabilize the crystal structure.

## Related literature

For related literature, see: Dhal *et al.* (1975 ▶); Fahrni *et al.* (2003 ▶); Kimura *et al.* (1977 ▶); Lombardino & Ottemes (1981 ▶); Manna *et al.* (2002 ▶); Rawal *et al.* (1963 ▶).
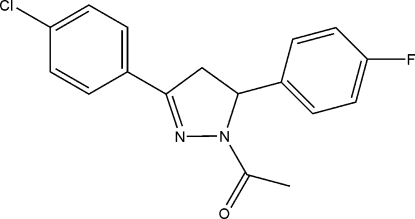


## Experimental

### 

#### Crystal data

C_17_H_14_ClFN_2_O
*M* *_r_* = 316.75
Monoclinic, 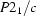

*a* = 14.5425 (19) Å
*b* = 11.3580 (14) Å
*c* = 9.6494 (13) Å
β = 108.154 (2)°
*V* = 1514.5 (3) Å^3^
*Z* = 4
Mo *K*α radiation
μ = 0.27 mm^−1^
*T* = 273 (2) K
0.14 × 0.12 × 0.06 mm

#### Data collection

Bruker SMART CCD area-detector diffractometer
Absorption correction: none
7793 measured reflections
2676 independent reflections
2077 reflections with *I* > 2σ(*I*)
*R*_int_ = 0.021

#### Refinement

*R*[*F* ^2^ > 2σ(*F* ^2^)] = 0.037
*wR*(*F* ^2^) = 0.098
*S* = 1.03
2676 reflections
200 parameters
H-atom parameters constrained
Δρ_max_ = 0.20 e Å^−3^
Δρ_min_ = −0.21 e Å^−3^

### 

Data collection: *SMART* (Bruker, 1997 ▶); cell refinement: *SAINT* (Bruker, 1997 ▶); data reduction: *SAINT*; program(s) used to solve structure: *SHELXS97* (Sheldrick, 2008 ▶); program(s) used to refine structure: *SHELXL97* (Sheldrick, 2008 ▶); molecular graphics: *SHELXTL* (Sheldrick, 2008 ▶); software used to prepare material for publication: *SHELXTL*.

## Supplementary Material

Crystal structure: contains datablocks global, I. DOI: 10.1107/S1600536808018333/at2570sup1.cif

Structure factors: contains datablocks I. DOI: 10.1107/S1600536808018333/at2570Isup2.hkl

Additional supplementary materials:  crystallographic information; 3D view; checkCIF report

## Figures and Tables

**Table 1:** Hydrogen-bond geometry (Å, °)

*D*—H⋯*A*	*D*—H	H⋯*A*	*D*⋯*A*	*D*—H⋯*A*
C4—H4*B*⋯O1^i^	0.97	2.57	3.425 (2)	147

Symmetry code: (i) 
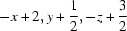
.

**Fig. 1.**
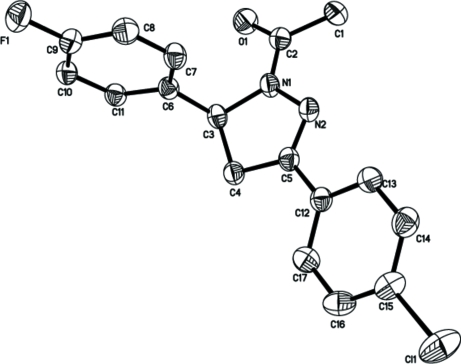
The molecular structure and atom-labeling scheme for (I), with displacement ellipsoids drawn at the 30% probability level.

**Fig. 2.**
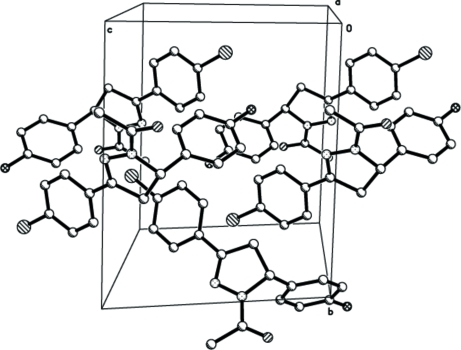
View of the crysytal packing of (I) in the unit cell.

## supplementary crystallographic information

### Comment

Pyrazoline and some of its derivatives demonstrate antiviral (Rawal *et al.*, 1963), antifungal (Dhal *et al.*, 1975), and immunosuppressive (Lombardino & Ottemes, 1981) activities. 1-Acetyl-3,5-diaryl-2-pyrazolines have been found to inhibit monoamine oxidases (Manna *et al.*, 2002). As part of our ongoing investigation of pyrazolines and their metal complexes, we report here the crystal structure of the title compound (I).

In (I) (Fig. 1), all bond lengths and angles are normal (Fahrni *et al.*, 2003; Kimura *et al.*, 1977). The mean plane of pyrazoline ring makes dihedral angles of 18.19 (1)° and 83.51 (4)° with 4-chlorobenzene ring and 4-fluorolbenzene ring, respectively. The dihedral angle between the two benzene rings is 76.11 (2)°. Weak intermolecular C—H···O hydrogen bonds help stabilize the crystal structure (Table 1). The crystal packing of (I) is shown in Fig. 2.

### Experimental

1-(4-chlorophenyl)-3-(4-fluorophenyl)-2-propenyl-1-ketone (0.02 mol)and hydrazine (0.02 mol)were mixed in 99.5% acetic acid (40 ml) and stirred in refluxing for 6 h, then the mixture was poured into ice-water to afford colourless solids.The solids were filtrated and washed with water until the pH of solution is about to 7.0. Finally, the solid crystals were dry under room temperature. Single crystals of the title compound suitable for X-ray measurements were obtained by recrystallization from EtOH at room temperature.

### Refinement

H atoms were fixed geometrically and allowed to ride on their parent atoms, with C—H distances of 0.93–0.976 Å, and with *U*_iso_=1.2–1.5*U*_eq_ of the parent atoms.

### Crystal data

**Table:**

C_17_H_14_ClFN_2_O	*F*_000_ = 656
*M**_r_* = 316.75	*D*_x_ = 1.389 Mg m^−^^3^
Monoclinic, *P*2_1_/*c*	Mo *K*α radiation λ = 0.71073 Å
Hall symbol: -P 2ybc	Cell parameters from 2887 reflections
*a* = 14.5425 (19) Å	θ = 2.9–25.9º
*b* = 11.3580 (14) Å	µ = 0.27 mm^−^^1^
*c* = 9.6494 (13) Å	*T* = 273 (2) K
β = 108.154 (2)º	Bar, colourless
*V* = 1514.5 (3) Å^3^	0.14 × 0.12 × 0.06 mm
*Z* = 4	

### Data collection

**Table:**

Bruker SMART CCD area-detector diffractometer	2077 reflections with *I* > 2σ(*I*)
Radiation source: fine-focus sealed tube	*R*_int_ = 0.021
Monochromator: graphite	θ_max_ = 25.1º
*T* = 273(2) K	θ_min_ = 2.3º
φ and ω scans	*h* = −17→17
Absorption correction: none	*k* = −13→11
7793 measured reflections	*l* = −11→9
2676 independent reflections	

### Refinement

**Table:**

Refinement on *F*^2^	Hydrogen site location: inferred from neighbouring sites
Least-squares matrix: full	H-atom parameters constrained
*R*[*F*^2^ > 2σ(*F*^2^)] = 0.037	*w* = 1/[σ^2^(*F*_o_^2^) + (0.0385*P*)^2^ + 0.4849*P*] where *P* = (*F*_o_^2^ + 2*F*_c_^2^)/3
*wR*(*F*^2^) = 0.098	(Δ/σ)_max_ < 0.001
*S* = 1.03	Δρ_max_ = 0.20 e Å^−^^3^
2676 reflections	Δρ_min_ = −0.21 e Å^−^^3^
200 parameters	Extinction correction: SHELXL97 (Sheldrick, 2008), Fc^*^=kFc[1+0.001xFc^2^λ^3^/sin(2θ)]^-1/4^
Primary atom site location: structure-invariant direct methods	Extinction coefficient: 0.0031 (10)
Secondary atom site location: difference Fourier map	

### Special details

**Table:**

Geometry. All e.s.d.'s (except the e.s.d. in the dihedral angle between two l.s. planes) are estimated using the full covariance matrix. The cell e.s.d.'s are taken into account individually in the estimation of e.s.d.'s in distances, angles and torsion angles; correlations between e.s.d.'s in cell parameters are only used when they are defined by crystal symmetry. An approximate (isotropic) treatment of cell e.s.d.'s is used for estimating e.s.d.'s involving l.s. planes.
Refinement. Refinement of *F*^2^ against ALL reflections. The weighted *R*-factor *wR* and goodness of fit *S* are based on *F*^2^, conventional *R*-factors *R* are based on *F*, with *F* set to zero for negative *F*^2^. The threshold expression of *F*^2^ > σ(*F*^2^) is used only for calculating *R*-factors(gt) *etc*. and is not relevant to the choice of reflections for refinement. *R*-factors based on *F*^2^ are statistically about twice as large as those based on *F*, and *R*- factors based on ALL data will be even larger.

### Fractional atomic coordinates and isotropic or equivalent isotropic displacement parameters (Å^2^)

**Table:**

	*x*	*y*	*z*	*U*_iso_*/*U*_eq_	
Cl1	0.45884 (5)	0.34606 (6)	0.02764 (8)	0.0949 (3)	
F1	0.70769 (11)	−0.14714 (11)	1.10898 (14)	0.0851 (4)	
O1	1.00066 (10)	−0.13914 (13)	0.72130 (15)	0.0685 (4)	
N1	0.88095 (10)	−0.02205 (13)	0.59652 (15)	0.0491 (4)	
N2	0.80952 (10)	0.00960 (13)	0.46876 (15)	0.0477 (4)	
C1	0.93307 (15)	−0.17908 (18)	0.4668 (2)	0.0610 (5)	
H1A	0.9661	−0.1361	0.4109	0.092*	
H1B	0.8660	−0.1873	0.4111	0.092*	
H1C	0.9617	−0.2557	0.4898	0.092*	
C2	0.94144 (13)	−0.11419 (16)	0.6044 (2)	0.0494 (4)	
C3	0.88134 (13)	0.05353 (16)	0.72122 (18)	0.0477 (4)	
H3	0.9474	0.0797	0.7730	0.057*	
C4	0.82018 (14)	0.15743 (16)	0.64155 (19)	0.0500 (5)	
H4A	0.7728	0.1808	0.6881	0.060*	
H4B	0.8604	0.2246	0.6373	0.060*	
C5	0.77233 (12)	0.10719 (16)	0.49247 (18)	0.0454 (4)	
C6	0.83785 (12)	−0.00700 (15)	0.82540 (18)	0.0446 (4)	
C7	0.76197 (13)	−0.08590 (17)	0.7766 (2)	0.0530 (5)	
H7	0.7404	−0.1070	0.6786	0.064*	
C8	0.71794 (15)	−0.13366 (18)	0.8711 (2)	0.0593 (5)	
H8	0.6671	−0.1868	0.8382	0.071*	
C9	0.75105 (15)	−0.10081 (18)	1.0151 (2)	0.0574 (5)	
C10	0.82613 (14)	−0.02457 (17)	1.0681 (2)	0.0537 (5)	
H10	0.8474	−0.0044	1.1664	0.064*	
C11	0.86977 (13)	0.02200 (16)	0.97206 (18)	0.0481 (4)	
H11	0.9215	0.0737	1.0065	0.058*	
C12	0.69236 (12)	0.16303 (16)	0.37998 (19)	0.0455 (4)	
C13	0.63734 (14)	0.10150 (18)	0.2576 (2)	0.0557 (5)	
H13	0.6499	0.0222	0.2473	0.067*	
C14	0.56443 (15)	0.15720 (19)	0.1515 (2)	0.0621 (5)	
H14	0.5275	0.1154	0.0703	0.075*	
C15	0.54634 (13)	0.27427 (18)	0.1657 (2)	0.0588 (5)	
C16	0.59780 (14)	0.33572 (18)	0.2860 (3)	0.0630 (6)	
H16	0.5842	0.4147	0.2957	0.076*	
C17	0.67020 (14)	0.28007 (17)	0.3936 (2)	0.0558 (5)	
H17	0.7046	0.3218	0.4764	0.067*	

### Atomic displacement parameters (Å^2^)

**Table:**

	*U*^11^	*U*^22^	*U*^33^	*U*^12^	*U*^13^	*U*^23^
Cl1	0.0695 (4)	0.0900 (5)	0.1055 (5)	0.0056 (3)	−0.0012 (3)	0.0364 (4)
F1	0.1184 (11)	0.0792 (9)	0.0750 (9)	−0.0204 (8)	0.0552 (8)	0.0010 (7)
O1	0.0622 (9)	0.0758 (10)	0.0578 (9)	0.0125 (7)	0.0045 (7)	0.0014 (7)
N1	0.0505 (8)	0.0581 (9)	0.0370 (8)	0.0056 (7)	0.0111 (7)	−0.0052 (7)
N2	0.0485 (8)	0.0565 (9)	0.0372 (8)	0.0015 (7)	0.0121 (6)	−0.0026 (7)
C1	0.0680 (13)	0.0577 (12)	0.0615 (12)	0.0051 (10)	0.0261 (10)	−0.0078 (10)
C2	0.0464 (10)	0.0528 (11)	0.0501 (11)	−0.0025 (9)	0.0167 (9)	0.0005 (9)
C3	0.0456 (10)	0.0571 (11)	0.0393 (9)	−0.0055 (8)	0.0115 (8)	−0.0086 (8)
C4	0.0573 (11)	0.0502 (11)	0.0437 (10)	−0.0045 (9)	0.0177 (8)	−0.0045 (8)
C5	0.0474 (10)	0.0504 (11)	0.0413 (9)	−0.0051 (8)	0.0180 (8)	−0.0026 (8)
C6	0.0431 (9)	0.0497 (10)	0.0381 (9)	0.0034 (8)	0.0084 (7)	−0.0024 (8)
C7	0.0529 (11)	0.0613 (12)	0.0416 (10)	−0.0062 (9)	0.0098 (8)	−0.0067 (9)
C8	0.0599 (12)	0.0570 (12)	0.0623 (13)	−0.0115 (10)	0.0209 (10)	−0.0044 (10)
C9	0.0710 (13)	0.0546 (12)	0.0542 (12)	0.0017 (10)	0.0302 (10)	0.0049 (9)
C10	0.0666 (12)	0.0541 (11)	0.0388 (10)	0.0037 (10)	0.0142 (9)	−0.0018 (8)
C11	0.0487 (10)	0.0498 (10)	0.0416 (10)	0.0001 (8)	0.0078 (8)	−0.0043 (8)
C12	0.0452 (10)	0.0504 (11)	0.0441 (10)	−0.0035 (8)	0.0188 (8)	0.0003 (8)
C13	0.0609 (12)	0.0519 (11)	0.0513 (11)	−0.0008 (9)	0.0131 (9)	−0.0012 (9)
C14	0.0592 (12)	0.0677 (14)	0.0526 (12)	−0.0076 (10)	0.0075 (10)	0.0003 (10)
C15	0.0450 (11)	0.0608 (13)	0.0685 (13)	−0.0036 (9)	0.0144 (9)	0.0141 (10)
C16	0.0534 (12)	0.0488 (11)	0.0880 (16)	0.0000 (9)	0.0238 (11)	0.0063 (11)
C17	0.0511 (11)	0.0532 (12)	0.0636 (12)	−0.0058 (9)	0.0183 (10)	−0.0062 (10)

### Geometric parameters (Å, °)

**Table:**

Cl1—C15	1.733 (2)	C6—C7	1.385 (2)
F1—C9	1.361 (2)	C7—C8	1.378 (3)
O1—C2	1.220 (2)	C7—H7	0.9300
N1—C2	1.354 (2)	C8—C9	1.373 (3)
N1—N2	1.3893 (19)	C8—H8	0.9300
N1—C3	1.477 (2)	C9—C10	1.362 (3)
N2—C5	1.285 (2)	C10—C11	1.381 (3)
C1—C2	1.490 (3)	C10—H10	0.9300
C1—H1A	0.9600	C11—H11	0.9300
C1—H1B	0.9600	C12—C17	1.384 (3)
C1—H1C	0.9600	C12—C13	1.391 (3)
C3—C6	1.510 (2)	C13—C14	1.377 (3)
C3—C4	1.533 (3)	C13—H13	0.9300
C3—H3	0.9800	C14—C15	1.371 (3)
C4—C5	1.502 (2)	C14—H14	0.9300
C4—H4A	0.9700	C15—C16	1.362 (3)
C4—H4B	0.9700	C16—C17	1.380 (3)
C5—C12	1.465 (2)	C16—H16	0.9300
C6—C11	1.385 (2)	C17—H17	0.9300
			
C2—N1—N2	122.87 (14)	C8—C7—H7	119.5
C2—N1—C3	124.48 (15)	C6—C7—H7	119.5
N2—N1—C3	112.62 (14)	C9—C8—C7	118.28 (18)
C5—N2—N1	107.76 (14)	C9—C8—H8	120.9
C2—C1—H1A	109.5	C7—C8—H8	120.9
C2—C1—H1B	109.5	F1—C9—C10	118.61 (18)
H1A—C1—H1B	109.5	F1—C9—C8	118.66 (19)
C2—C1—H1C	109.5	C10—C9—C8	122.73 (18)
H1A—C1—H1C	109.5	C9—C10—C11	118.23 (17)
H1B—C1—H1C	109.5	C9—C10—H10	120.9
O1—C2—N1	119.36 (17)	C11—C10—H10	120.9
O1—C2—C1	123.17 (18)	C10—C11—C6	121.12 (17)
N1—C2—C1	117.46 (17)	C10—C11—H11	119.4
N1—C3—C6	112.45 (15)	C6—C11—H11	119.4
N1—C3—C4	100.64 (13)	C17—C12—C13	118.28 (18)
C6—C3—C4	112.75 (14)	C17—C12—C5	120.02 (17)
N1—C3—H3	110.2	C13—C12—C5	121.70 (17)
C6—C3—H3	110.2	C14—C13—C12	120.43 (19)
C4—C3—H3	110.2	C14—C13—H13	119.8
C5—C4—C3	102.19 (14)	C12—C13—H13	119.8
C5—C4—H4A	111.3	C15—C14—C13	119.92 (19)
C3—C4—H4A	111.3	C15—C14—H14	120.0
C5—C4—H4B	111.3	C13—C14—H14	120.0
C3—C4—H4B	111.3	C16—C15—C14	120.72 (19)
H4A—C4—H4B	109.2	C16—C15—Cl1	119.42 (17)
N2—C5—C12	121.50 (16)	C14—C15—Cl1	119.84 (17)
N2—C5—C4	113.79 (16)	C15—C16—C17	119.62 (19)
C12—C5—C4	124.69 (16)	C15—C16—H16	120.2
C11—C6—C7	118.63 (17)	C17—C16—H16	120.2
C11—C6—C3	119.71 (16)	C16—C17—C12	120.97 (19)
C7—C6—C3	121.53 (15)	C16—C17—H17	119.5
C8—C7—C6	120.99 (17)	C12—C17—H17	119.5

### Hydrogen-bond geometry (Å, °)

**Table:**

*D*—H···*A*	*D*—H	H···*A*	*D*···*A*	*D*—H···*A*
C4—H4B···O1^i^	0.97	2.57	3.425 (2)	147

Symmetry codes: (i) −*x*+2, *y*+1/2, −*z*+3/2.
